# SFRP1 Expression is Inversely Associated With Metastasis Formation in Canine Mammary Tumours

**DOI:** 10.1007/s10911-023-09543-z

**Published:** 2023-07-04

**Authors:** Judith Seitz, Alan Bilsland, Chloé Puget, Ian Baasner, Robert Klopfleisch, Torsten Stein

**Affiliations:** 1 Institute of Veterinary Biochemistry, Department of Veterinary Medicine, Freie Universität Berlin, Berlin, Germany; 2 Wolfson Wohl Cancer Research Centre, Institute of Cancer Sciences, College of MVLS, University of Glasgow, Glasgow, UK; 3 Institute of Veterinary Pathology, Department of Veterinary Medicine, Freie Universität Berlin, Berlin, Germany

**Keywords:** Canine Mammary Tumours, Metastasis, SFRP1, RNA Signature

## Abstract

**Background:**

Canine mammary tumours (CMTs) are the most frequent tumours in intact female dogs and show strong similarities with human breast cancer. In contrast to the human disease there are no standardised diagnostic or prognostic biomarkers available to guide treatment. We recently identified a prognostic 18-gene RNA signature that could stratify human breast cancer patients into groups with significantly different risk of distant metastasis formation. Here, we assessed whether expression patterns of these RNAs were also associated with canine tumour progression.

**Method:**

A sequential forward feature selection process was performed on a previously published microarray dataset of 27 CMTs with and without lymph node (LN) metastases to identify RNAs with significantly differential expression to identify prognostic genes within the 18-gene signature. Using an independent set of 33 newly identified archival CMTs, we compared expression of the identified prognostic subset on RNA and protein basis using RT-qPCR and immunohistochemistry on FFPE-tissue sections.

**Results:**

While the 18-gene signature as a whole did not have any prognostic power, a subset of three RNAs: *Col13a1*, *Spock2*, and *Sfrp1*, together completely separated CMTs with and without LN metastasis in the microarray set. However, in the new independent set assessed by RT-qPCR, only the Wnt-antagonist *Sfrp1* showed significantly increased mRNA abundance in CMTs without LN metastases on its own (*p* = 0.013) in logistic regression analysis. This correlated with stronger SFRP1 protein staining intensity of the myoepithelium and/or stroma (*p* < 0.001). SFRP1 staining, as well as β-catenin membrane staining, was significantly associated with negative LN status (*p* = 0.010 and 0.014 respectively). However, SFRP1 did not correlate with β-catenin membrane staining (*p* = 0.14).

**Conclusion:**

The study identified SFRP1 as a potential biomarker for metastasis formation in CMTs, but lack of SFRP1 was not associated with reduced membrane-localisation of β-catenin in CMTs.

**Supplementary Information:**

The online version contains supplementary material available at 10.1007/s10911-023-09543-z.

## Introduction

Mammary tumours are the most prevalent neoplasms in intact female dogs with approximately half of these tumours being malignant [1], typically leading to death through metastasis formation within two years of diagnosis [2].

Although canine mammary tumours (CMTs) show many similarities with the human disease, in contrast to breast cancer there are currently no standardised diagnostic or prognostic biomarkers available in dogs to guide treatment [3]. Diagnosis relies mostly on histological classification [4], and tumour types differ more extensively than in humans, with more mixed/complex tumours containing a myoepithelial as well as an epithelial component [5, 6]. Histological subtype is related to outcome [7]; however, methods of tumour classification can vary considerably despite efforts to standardise diagnostic procedures [8]. Treatment options for mammary carcinomas are also most often limited to surgical removal of the diseased gland(s) as the evidence that chemotherapy has proven survival benefit is limited due to small study size and variation in histological types and clinical stage, and no targeted therapies are routinely available [3, 9]. Therefore, there is not only a clinical need for a reliable prognostic biomarker to guide veterinary oncologists and owners towards the best treatment for their pets, but also a need for new effective treatment options [10].

It has long been established that molecular pathways implicated in normal mammary gland morphogenesis also play critical roles during breast cancer progression [11, 12]. To better understand the processes and mechanisms involved in the invasion and spreading of mammary cancer cells our lab has therefore been studying the molecular changes that occur during normal mammary gland development [13–15]. For instance, the histological changes that occur during puberty- and pregnancy-induced mammary branching morphogenesis and cancer invasion show distinct similarities, so that this normal physiological process could be described as a type of ‘controlled invasion’. Particularly during pregnancy milk ducts form lateral side branches and alveoli, a process requiring remodelling of the surrounding extracellular matrix (ECM), including breakdown of existing basement membrane (BM)/collagen sheath and formation of a new BM and collagen network [16]. Our lab has recently identified a fibroblast-derived signature of matrisome-encoding genes (‘matriscore’) based on changes that occur during very early pregnancy-associated branching morphogenesis in mice [17]. This RNA signature of 18 differentially expressed genes was able to significantly stratify human breast cancer patients of two large microarray datasets into groups with a high or low chance of distant metastasis-free survival (DMFS) and recurrence-free survival (RFS) over 10 years [18] in the presence of other clinical parameters, including grade, size, age, as well as lymph node (LN)- and oestrogen receptor (ER) status.

In this study, we have attempted to use our knowledge from human breast cancer to identify a potential biomarker for metastasis formation in CMTs. Here we show that a subgroup of just three of the 18 genes: collagen XIII, alpha 1 (*Col13a1*), secreted frizzled-related protein 1 (*Sfrp1*), and sparc/osteonectin, cwcv and kazal-like domains proteoglycan (testican) 2 (*Spock2*), was able to stratify a previously described canine mammary cancer cohort of 27 ER_neg_ patients [19] into groups of mammary tumours with Met_pos_ and Met_neg_ status, and hence poor or good survival over 24 months. Using RT-qPCR and IHC on another independent cohort of 33 randomly selected formalin-fixed, paraffin-embedded (FFPE) CMT samples, we established that reduced mRNA and protein abundance of the suppressor of Wnt-signalling *Sfrp1* correlated significantly with metastasis. Consistent with this finding, staining for β-catenin showed that reduced membrane staining, indicative of canonical Wnt-pathway activation, also correlated significantly with metastasis status. However, there was no significant correlation between SFRP1 expression and β-catenin membrane localisation. Our study has therefore established for the first time that reduced expression of SFRP1 is significantly associated with metastasis formation in canine mammary cancer. However, a reduction in SFRP1 expression does not appear to be associated with canonical Wnt-pathway activation in our cohort.

## Material and Methods

### Data Analysis

To identify mRNAs in our 18 gene signature that could contribute significantly to the stratification of the 27 CMT cohort into LN_pos_ and LN_neg_ groups, multivariate logistic regression for presence/absence of metastasis was used in the microarray dataset of Klopfleisch et al. [19] using Firth’s bias-reduced method [20]. The model was fitted using the logistf function in R package version 1.24.1. (https://CRAN.R-project.org/package=logistf). Confidence intervals and *p*-values were calculated using profile likelihood [21]. Univariate logistic regression was used to assess *Spock2*, *Col13a1*, and *Sfrp1* values analysed by RT-qPCR in relation to presence of metastasis.

Similarly, logistic regression was used to assess SFRP1 staining and β-catenin membrane staining scores (negative (0), weak (1–6), moderate (8–12) or strong (> 12)) in relation to metastasis, while correlation between *Sfrp1* mRNA abundance (0: < 1%; 1: 1–10%; 2: 11–25%; 3: > 25% of *Rps19* mRNA abundance), SFRP1 and β-catenin protein staining scores, and grade (1–3) was analysed using the correlation analysis function in SPSS.

### Tissues

Residual archival material of formalin-fixed paraffin-embedded (FFPE) mammary tissue that had been sent to the Institute of Veterinary Pathology for diagnostic purposes between 2016 and 2021 was used. Canine mammary gland tumours were graded by C. Puget and R. Klopfleisch based on the grading system according to M. Goldschmidt et al. [4] and assessed histologically for lymphatic vessel metastases (N0-N1) and lymph node metastases (M0-M1). Surrounding morphologically normal canine mammary tissue was used as ‘healthy’ controls.

### RNA Isolation

Five 10 µm sections of formalin-fixed paraffin-embedded tissue-blocks were cut with a microtome and collected per tissue sample. Three isolates were produced from each block. Blades were cleaned and treated with RNAse away (ThermoFisher Scientific, Waltham, MA, USA) between blocks.

RNA was isolated using the NucleoSpin® totalRNA FFPE XS kit (Macherey & Nagel, Düren, Germany) according to the manufacturer’s protocol, including on-column DNase treatment.

RNA was eluted in 15 μl RNase-free H_2_O and stored at -20 °C for 24 h or at -70 °C for long term storage. Concentration and purity were assessed using a NanoDrop ND-1000 (peqLab Biotechnology GmbH, Erlangen, Germany).

### RT-qPCR

cDNAs were synthesised using the LunaScriptRT® SuperMix Kit (NEB, Frankfurt/Main, Germany), containing random hexamers as well as poly-dT primers according to the manufacturer’s protocol in an Mastercycler Gradient thermocycler (Eppendorf, Hamburg, Germany) to avoid 3’-5’ bias and enable even reverse transcription along the whole mRNA. 250 ng total RNA from the FFPE tissue sections was used for each reaction. A negative control without reverse transcriptase was prepared for each sample. cDNAs were frozen and stored at -20 °C until further use.

Primers and LNA-containing dual-labelled probes (6-FAM, BHQ1) (Supplementary Table 1) were designed using the Sigma OligoArchitect™ Online (Merck, Darmstadt, Germany) and Tm Prediction (Qiagen, Hilden, Germany) webtools, and produced by Sigma-Aldrich (Merck). The primers used were intron spanning to avoid genomic DNA amplification and all amplicons were between 70 and 110 bp in size to enhance chances of amplification in potentially degraded RNA.qPCR was performed using the Luna Universal Probe qPCR MasterMix (NEB) in 10 µl reactions within a StepOne™ Thermal Cycler (Applied Biosystems, Waltham, MA, USA). Each cDNA was diluted 1:5 before use and each reaction performed in triplicate. No-RT and H_2_O-only negative controls were used for each sample. Primers and probes were used at 0.4 µM. Cycles used were: activation at 95 °C for 60 s followed by 45 cycles of 95 °C for 15 s, 60 °C for 30 s. Ct-values for each RNA were normalised against *Rps19* and presented as ‘% of *Rps19*’ by dividing 100 by the 2^−ΔCt^ value for each sample. This way of presentation maintains the relative differences between each sample as would be seen by the classical ΔΔCt method, where one sample acts as the ‘control’ to create a ΔΔCt, while additionally showing the detection level relative to the house-keeping gene (*Rps19*) and thereby emphasising high or low detection. Mean values were calculated for each triplicate sample.

### IHC

All histological sections were prepared and stained within the Institute of Veterinary Pathology. 2–4 μm sections were collected on a cold-water bath (20 °C), stretched on a hot water bath (45 °C) and carefully applied onto an adhesion (silanised) microscope slide. The mounted sections were then dried overnight at 37 °C. Sections were dewaxed by incubation in xylene twice for 10 min, then twice in 100% ethanol for 3 min, twice for 3 min in 96% ethanol and once for 3 min in 70% ethanol. For antigen retrieval, sections were pre-treated with citrate buffer (β-catenin) and Tris/EDTA, pH9 (SFRP1) respectively for 12 min at 600Watt in a microwave, cooled and washed three times in PBS. Blocking was performed using normal goat serum (Biowest, Nuaillé, France) diluted 1:5 for 30 min at RT. Primary antibodies rabbit anti-SFRP1 mAb (ab126613; Abcam; Berlin, Germany) and mouse anti-β-catenin (clone 14; 610,154; BD Biosciences, Heidelberg, Germany) were applied at a dilution of 1:200 in PBS/BSA 2% and incubated overnight at 4 °C. Normal rabbit Ig diluted in PBS was used as a negative control except for anti-β-catenin staining, where normal mouse Ig diluted in PBS was used. Following three washes, secondary antibody (biotinylated goat anti-rabbit IgG (1:200) or goat anti-mouse (1:200) in PBS/goat-normal serum; VEC-BA-1000 / VEC-BA-9200; Vector Laboratories, Inc.) was applied for 30 min at RT. After another three washes, the avidin–biotin-peroxidase complex (ABC) solution of the Vectastain® ABC-Elite-Kit PK 6100 (Vector Laboratories, Inc.) was added for 30 min at RT. DAB solution was added for 5 min at RT for staining. Afterwards, the sections were washed three times with dH_2_O and nuclei counterstained with haematoxylin. The sections were again dehydrated using an increasing alcohol series and xylene. The slides were mounted in mounting media (Vector Laboratories, Inc) and scanned with an Aperio CS2 ScanScope Slide Scanner (Leica Biosystems, Wetzlar, Germany) at 40X magnification. The blocks were categorised according to their IHC staining into no, weak, moderate and strong staining and scored according to the “Quickscore” method [22].

#### Western Blot

Canine mammary epithelial MTH53A cells were transfected with 1 µg of pCMV-Sfrp1 (SFRP1_OFb05324C_pcDNA3.1( +) SC1200 Clone; Genscript Biotech, Rijswijk, Netherlands) encoding feline SFRP1 using TransIT®-LT1 Transfection Reagent (Mirus, Madison; WI, USA) or without plasmid. Total protein was extracted after 24 h using RIPA buffer, quantified by Pierce™ BCA Protein Assay (Thermo Fisher Scientific) and 25 µg separated on a 10% Bis–Tris SDS–polyacrylamide gel (Merck, Darmstadt, Germany) using MOPS running buffer. Proteins were transferred onto nitrocellulose by semi-dry blot and transfer confirmed by Ponceau-S staining. Membranes were blocked in TBST containing 3% fat-free milk powder and incubated with rabbit anti-SFRP1 (1:1000, ab126613, Abcam) in blocking buffer overnight at 4 °C. Bound antibody was detected using a HRP-linked donkey anti-rabbit antibody (1:10,000, NA934-1ML, Cytiva, Freiburg, Germany). Chemiluminescence was detected using a Cytiva Amersham™ ECL Prime Western Blotting Detection Reagent (Thermo Fisher Scientific) and a Fusion SL imager (Vilbert Lourmat, Eberhardzell, Germany). Membranes were stripped using a 0.1% SDS/200 mM glycine buffer, pH2.2, and subsequently incubated with a mouse anti-β-actin antibody (1:10,000, 66009–1-Ig, Proteintech, Manchester, UK), followed by HRP-linked sheep anti-mouse antibody (1:10,000, NA931-1ML, Cytiva) and Amersham™ ECL Select™ Western Blotting Detection Reagent as described above.

## Results

### Expression of *Col13a1*, *Spock2*, and *Sfrp1* Negatively Correlates with LN-status

In humans, the 18 gene signature was able to stratify breast cancer cohorts into groups with high or low risk of DMFS. To test whether these results were translatable to the dog, we used the same method as previously described [17] on a RNA microarray dataset from a cohort of 27 canine mammary cancers (CMCs) [19]. Contrary to humans, the combined signature was unable to significantly distinguish between high and low risk (data not shown). To test whether a subset of these genes was associated with metastases we performed a sequential forward feature selection process using the ‘sequentialfs’ function in Matlab (Mathworks, Cambridge, UK).

This is an algorithm, in which each single feature (i.e. each of the 18 genes) is first tested to determine the best-fitting single-variable logistic regression model to separate cancers with positive and negative LN-status, which has been determined histologically previously [19]. In subsequent steps, remaining features are again tested one at a time in combination with variables already selected and these are retained one at a time if they significantly improved the goodness-of-fit of the logistic regression model. i.e. improved the separation. Goodness-of-fit measures quantify difference between observed and model-predicted outcomes. Sequential (or step-wise) feature selection strategies and goodness-of-fit measures for logistic regression are further discussed in Stoltzfus (2011) [23]. Individual variables were retained if they significantly improved model fit relative to a model lacking that variable assessed by chi-square test with one degree of freedom.

**Fig. 1:**
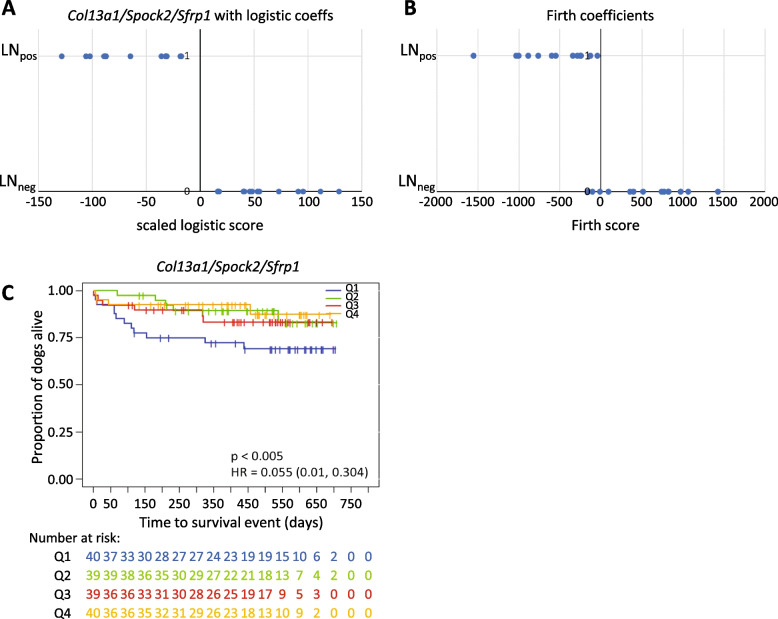
Complete separation of the 27 cases of canine mammary cancer based on the sum of expressions of *Col13a1*, *Spock2*, and *Sfrp1* mRNAs. **A** Lymph node positive cases (y-axis = 1) and negative cases (y-axis = 0) as defined by [19] were completely separated by the weighted sum of *Col13a1*, *Spock2*, and *Sfrp1* expression: -2.04 × *Col13a1* – 0.968 × *Sfrp1* – 1.27 × *Spock2*. These coefficients are scaled from those observed in standard logistic regression at the maximum iteration. As discussed in the main text and Table 1, Firth’s method of penalised logistic regression was subsequently employed to generate finite coefficient estimates. Group separation of tumours with (LN_pos_) and without metastases (LN_neg_), as defined in [19] with an alternative score using these coefficients is also shown (right panel) (**B**). **C** Kaplan–Meier survival analysis using RNA sequencing data from the Kim et al. [25] dataset of 158 CMTs showed that patients within the lowest expression quartile (Q1) for the three mRNAs had a significantly lower overall survival (*p* < 0.005)

This variable selection applied to the array dataset first retained *Spock2* and *Col13a1*. Subsequent addition of *Sfrp1* completely separated tumours on the basis of LN-status (Fig. 1). Figure 1A shows separation of LN_pos_/LN_neg_ cases by the selected model. Each point represents a case with LN status on the y-axis. X-axis values are the sum of expression values of the selected genes in each case, with values scaled by the respective coefficients for that gene in the model. However, perfect separation in a logistic regression model leads to a phenomenon in which maximum likelihood estimates of the coefficients cannot be obtained and the model fails to converge-an infinite number of curves parameterised by different regression coefficients would separate the outcomes equally well. In this case, lack of convergence is handled in a software-specific way and output coefficient estimates would depend on, for example, a maximum stopping iteration [24]. Nevertheless, likelihood ratio tests dropping each gene from the full model indicated that each term was independently highly significant (not shown). We therefore performed Firth’s bias-reduced method [20] of penalised logistic regression to obtain finite coefficient estimates (Fig. 1B). This approach adds a penalty term in the log likelihood function, which shrinks the coefficient estimates ensuring finite estimates. Classification performance of ‘canine matriscores’ based on both these analyses are shown in Fig. 1. As shown in Table 1, each gene remained highly significant (Table 1).

**Table 1:** Logistic regression analysis results for *Spock2*, *Col13a1*, and *Sfrp1* mRNA expression and metastasis status in the 27 CMTs from Klopfleisch et al. (2010) [19] as measured by microarray analysis using Firth’s penalised method

**Variable**	**B**	**SE**	***p*-value**	**exp{B}**	**95% profile CI for B**	
					**Lower**	**Upper**
***Spock2***	-3.194	1.278	< 0.001	0.041	-13.527	-1.106
***Col13a1***	-5.048	2.017	< 0.001	0.006	-18.311	-1.588
***Sfrp1***	-2.370	0.976	< 0.001	0.093	-10.427	-0.749

*B* logistic regression coefficient, *SE* standard error, *CI* confidence interval

**Table 2:** Univariate logistic regression analysis results for *Spock2*, *Col13a1*, and *Sfrp1* mRNA expression and metastasis status in the new independent cohort of 33 CMTs as measured by RT-qPCR analysis

**Variable**	**B**	**SE**	***p*-value**	**exp{B}**	**95% CI for exp{B}**	
					**Lower**	**Upper**
***Spock2***	0.176	0.501	0.726	1.192	0.447	3.183
***Col13a1***	0.719	1.533	0.639	2.052	0.102	41.417
***Sfrp1***	-0.122	0.049	0.013	0.885	0.804	0.974

*B* logistic regression coefficient, *SE* standard error, *CI* confidence interval

To test whether these results could be verified in an independent dataset, we analysed their expression in a published dataset from Kim et al. [25] containing RNA sequencing data from 158 CMTs. As LN-status had not been assessed in this study, lymph vessel (LV) invasion was used as a marker for cancer progression instead. Using this set, the three genes were unable to separate the LV_pos_ from the LV_neg_ groups (data not shown). However, Kaplan–Meier analysis confirmed that the quartile of patients with the lowest sum of *Sfrp1* mRNA expression had a significantly lower overall survival (*p* < 0.005; Fig. 1C).

### *Sfrp1* RNA Abundance is Associated With Negative Metastasis (Met_neg_) Status in CMTs

Because of the differing results in the two CMT datasets, we next asked how each of the three RNAs contributed to the stratification. We assessed each mRNA in an independent dataset of archival FFPE-tissues with known local metastasis status using RT-qPCR. A total of 24 metastatic and 23 non-metastatic canine mammary gland tumours were identified in the tissue archive. In addition, morphologically normal canine mammary tissues the canine mammary gland tumours were examined. All CMTs were again assessed for their metastatic appearance; i.e. invasion into lymphatic vessels and/or lymph nodes. Cases were dismissed if no lymphatic vessels were visible and no lymph nodes had been available from the corresponding dog. Further, all blocks that did not provide sufficient RNA were discarded, so that 38 blocks (17 metastatic, 16 non-metastatic, five morphologically normal canine mammary gland tissues) were further assessed (Supplementary Table 2). The 33 tumour samples were grouped into Met_pos_ and Met_neg_ based on the LV and LN invasion. The Met_pos_ group of 17 samples all showed LV invasion and 14 of these 17 also had a LN_pos_ status, while no lymph node information was available for the residual three (samples M10, M14, M17). Of the Met_neg_ group of 16 cases, 15 samples showed no LV invasion and 13 of these were also LN_neg_, while no lymph node information was available for the residual two samples. Lymphatic vessels were not visible in one sample (N3) but the corresponding local lymph node showed no metastases. The metastatic tumour blocks M2 and M16 blocks, as well as M15 and M5, were different tumours collected from the same two patients. All other canine mammary gland tumours came from individual patients.

**Fig. 2:**
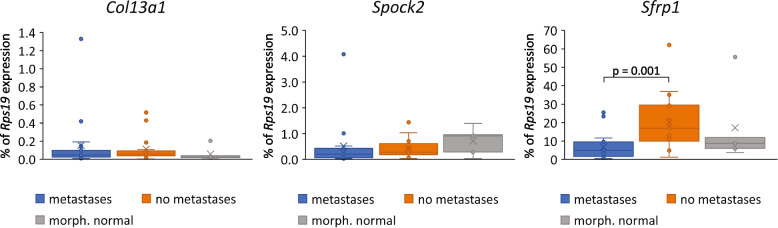
Comparison of RT-qPCR results for *Col13a1*, *Spock2*, and *Sfrp1* using total RNA isolated from FFPE-material from metastatic and non-metastatic CMTs, and morphologically normal canine mammary tissue*.* Box and whisker plots showing the mRNA abundance for *Col13a1, Spock2,* and *Sfrp1* as % of *Rps19* expression in CMTs with (metastasis, *n *= 17) and without (no metastasis; *n* = 16) tumour spread, as well as in morphologically normal mammary tissue (morph. normal; *n* = 5). Boxes define the interquartile range, with the median (horizontal line) and average expression (X) shown. Dots show individual outliers. Only *Sfrp1* showed a significant difference in abundance levels between CMTs with and without metastases in a Mann–Whitney U Test analysis (*p* = 0.001)

Expression was assessed relative to *Rps19* mRNA. The box-plots in Fig. 2 show the abundance of each RNA as % of *Rps19*. *Col13a1* showed a slightly higher abundance in CMTs compared to morphologically normal mammary tissue. *Spock2* showed a slightly reduced abundance in CMTs compared to morphologically normal control tissues, with CMTs with metastasis again having a slightly lower abundance compared to Met_neg_ tissues. However, neither reached statistical significance in a univariate logistic regression model. In contrast, *Sfrp1* abundance was higher in CMTs with Met_neg_ status compared to both CMTs with Met_pos_ status and morphologically normal tissue, with the former reaching statistical significance (*p* = 0.013) (Table 2). This indicated that in canine CMTs higher levels of *Sfrp1* mRNA as assessed by RT-qPCR was associated with a lower rate of progression.

### SFRP1 Protein Detection is Associated With Met_neg_ Status in CMTs

**Fig. 3:**
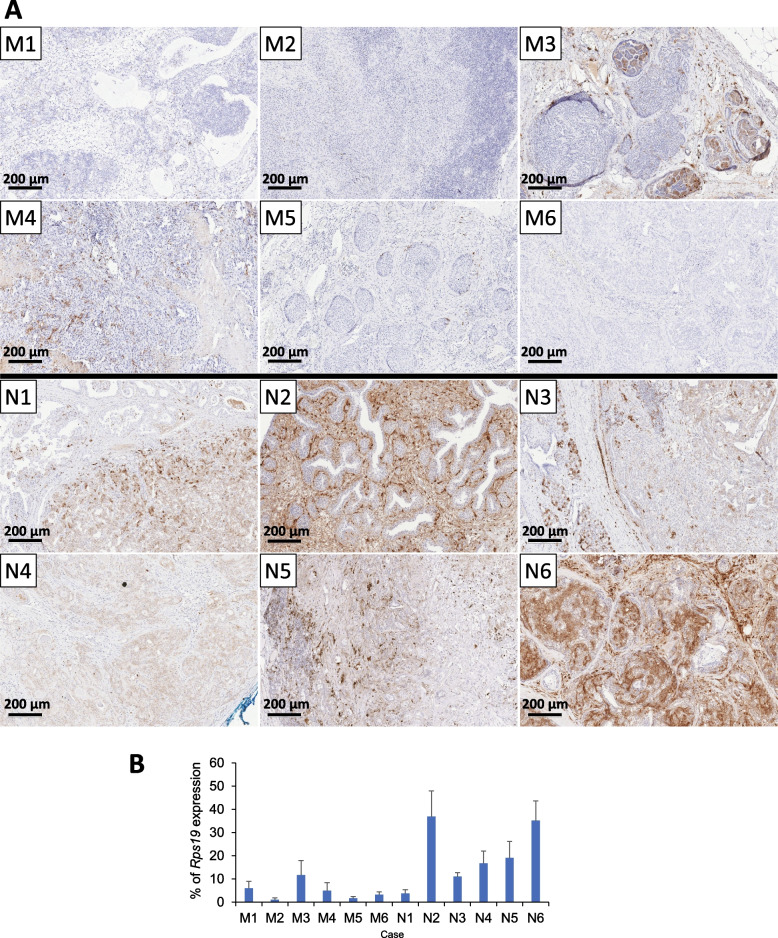
SFRP1 protein expression (IHC) in CMTs with and without metastasis. **A** Immunohistochemistry for SFRP1 in six randomly chosen examples of CMTs with (M1-6) and without (N1-6) metastasis compared to the mRNA expression levels (**B**) shown as % of *Rps19* expression (bars show standard deviation of replicates) of the same FFPE-blocks showing good correlation between protein and mRNA abundances. Bars represent 200 µm

To test whether this was also reflected on protein level, immunohistochemistry was performed on sections cut from the same blocks to assess SFRP1 expression in the same CMTs. As only Sfrp1mRNA was significantly associated with metastasis in the new independent dataset, we focussed only on SFRP1. Morphologically normal mammary tissue showed variable stromal and/or myoepithelial cell staining of SFRP1 protein within the TDLU, with individual stromal cells showing very strong signal intensities, while the luminal epithelium and major lactiferous ducts were mostly negative (Supplementary Fig. 1A). Staining intensities in tumour tissue correlated well with the relative mRNA expression levels (*p* < 0.001) (Fig. 3A + B, Supplementary Fig. 2). SFRP1 staining further correlated negatively with grade (*p* = 0.040) and Met status in a logistic regression analysis (*p* = 0.010), with Met_neg_ CMTs showing a stronger signal in stromal / myoepithelial cells surrounding the luminal epithelium, with only weak positivity within some malignant cells (see Fig. 3; N6). In contrast, CMTs with Met_pos_ status were mostly negative for SFRP1 with occasional weak or focal positivity (see Fig. 3; M3 + 4), and with strongest positivity found associated with histologically normal tissue around the tumour margins. These results are consistent with SFRP1’s known role in human breast cancer as a tumour suppressor and indicates that it may play a similar role in dogs.

### Changes in β-Catenin Localisation Do Not Correlate With Reduced SFRP1 Expression in CMTs

**Fig. 4:**
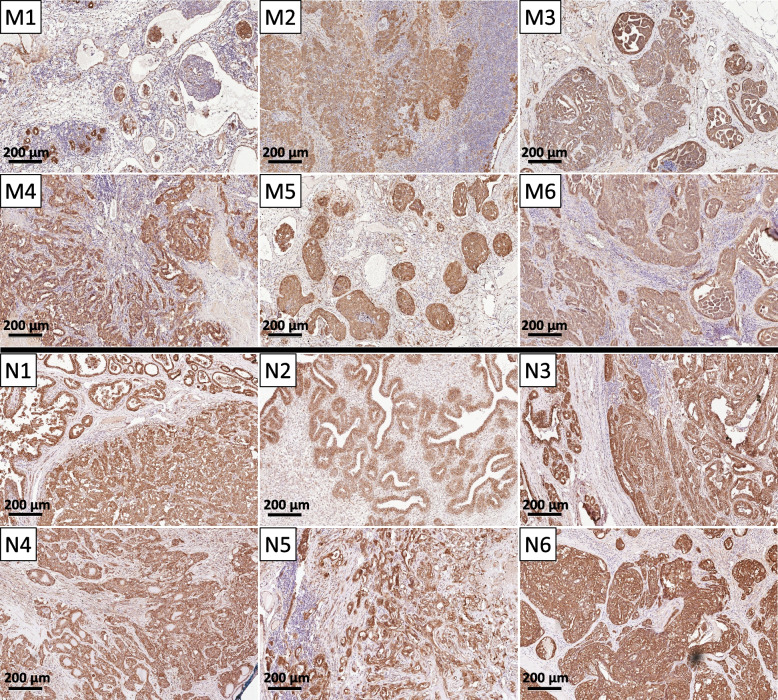
β-catenin protein expression (IHC) in CMTs with and without metastasis. **A** Immunohistochemistry for β-catenin in the same six examples of CMTs with (M1-6) and without (N1-6) metastasis as shown in Fig. 2. Membrane staining did correlate with metastasis status, but not with SFRP1 expression. Bars represent 200 µm

Since SFRP1 is a negative regulator of Wnt-signalling, we wanted to learn whether a reduction in SFRP1 abundance in CMTs correlated with canonical Wnt-pathway activation in our canine tumours. Based on a previous study in human breast cancer [26] we assessed changes in β-catenin membrane and nuclear localisation compared to surrounding morphologically normal mammary epithelium as a potential indicator of Wnt-pathway activation [27]. All morphologically normal mammary tissues from healthy individuals showed strong SFRP1 expression as well as membrane-associated staining for β-catenin with no nuclear positivity (Fig. 4, Supplementary Fig. 3). Similarly, CMTs with Met_neg_ status showed mostly strong membrane-associated β-catenin staining, while loss of membrane staining correlated negatively with grade (*p* = 0.005) and Met_neg_ status (*p* = 0.014; Table 3). In contrast, moderate to strong nuclear staining was only observed in three cases, with no correlation with metastasis status or SFRP1 staining. Similarly, loss of membrane-associated β-catenin did not correlate with reduced SFRP1 staining (*p* = 0.143); hence, there was no evidence that loss of SFRP1 has led to canonical Wnt-pathway activation in our dataset.

**Table 3:** Immunohistochemistry results for SFRP1 as well as membrane (m) or nuclear (n) associated β-catenin in the new cohort of 33 CMTs compared to *Sfrp1* mRNA expression (0–3), grade (1–3), and metastasis status (neg, pos)

	**SFRP1**					
	**Negative, n (%)**	**Weak, n (%)**	**Moderate, n (%)**	**Strong, n (%)**	**Total, n (%)**	***P***
***Sfrp1* mRNA**^**a**^						
0 (< 1%)	1 (16.7)	2 (28.6)	0 (0.0)	0 (0.0)	3 (21.4)	< 0.001
1 (1–10%)	5 (83.3)	4 (57.1)	4 (80.0)	1 (7.1)	14 (43.8)	
2 (11–25%)	0 (0.0)	1 (14.3)	1 (20.0%)	7 (50.0)	9 (28.1)	
3 (> 25%)	0 (0.0)	0 (0.0)	0 (0.0)	6 (42.9)	6 (18.6)	
**Grade**						
1	1 (16.7)	1 (14.3)	1 (20.0)	5 (35.8)	8 (25.0)	0.040
2	1 (16.7)	1 (14.3)	2 (40.0)	6 (42.9)	10 (31.3)	
3	4 (66.7)	5 (71.4)	2 (40.0)	3 (21.4)	14 (43.8)	
**Metastasis**						
Negative	1 (16.7)	1 (14.3)	3 (60.0)	10 (71.4)	15 (46.9)	0.010^b^
Positive	5 (83.3)	6 (85.7)	2 (40.0%)	4 (28.6)	17 (43.1)	
**β-Catenin (m)**						
Neg	1 (16.7)	1 (14.3)	0 (0.0)	2 (14.3)	4 (12.9)	0.143
Weak	4 (66.7)	3 (42.9)	2 (50.0)	4 (28.6)	16 (51.6)	
Moderate	1 (16.7)	2 (28.6)	0 (0.0)	4 (28.6)	4 (12.9)	
Strong	0 (0.0)	1 (14.3)	2 (50.0)	4 (28.6)	7 (22.6)	
**β-Catenin (n)**						
Neg	6 (16.7)	5 (83.3)	3 (75.0)	12 (85.7)	26 (83.9)	0.773
Weak	0 (0.0)	1 (16.7)	1 (25.0)	0 (0.0)	2 (6.5)	
Moderate	0 (0.0)	0 (0.0)	0 (0.0)	2 (14.3)	2 (6.5)	
Strong	0 (0.0)	1 (16.7)	0 (0.0)	0 (0.0)	1 (3.2)	
	**β-Catenin (m)**					
	**Negative, n (%)**	**Weak, n (%)**	**Moderate, n (%)**	**Strong, n (%)**	**Total, n (%)**	***P***
**Grade**						
1	0 (0.0)	1 (7.7)	3 (42.9)	4 (57.1)	8 (57.1)	0.005
2	2 (50.0)	4 (30.8)	0 (0.0)	3 (42.9)	9 (64.3)	
3	2 (50.0)	8 (61.5)	4 (57.1)	0 (0.0)	14 (45.2)	
**Metastasis**						
Negative	1 (25.0)	3 (23.1)	4 (57.1)	6 (85.7)	14 (45.2)	0.014^b^
Positive	3 (75.0)	10 (76.9)	3 (42.9)	1 (14.3)	17 (54.8)	
**SFRP1**						
Neg	1 (25.0)	4 (30.8)	1 (14.3)	0 (0.0)	6 (19.4)	0.143
Weak	1 (25.0)	3 (23.1)	2 (28.6)	1 (14.3)	9 (64.3)	
Moderate	0 (0.0)	2 (15.4)	0 (0.0)	2 (28.6)	2 (6.5)	
Strong	2 (50.0)	4 (30.8)	4 (57.1)	4 (57.1)	14 (45.2)	

^a^% of *Rps19* mRNA abundance

^b^Binary logistic regression

## Discussion

For many years, scientists have aimed to create tools for the accurate prognosis of canine mammary cancers. While the histological classifications including the WHO [28] and Goldschmidt et al. [4] classifications have both shown to be relatively good markers of cancer outcome, there have also been some conflicting results [2]. Therefore, additional prognostic markers that can assist veterinarians in their treatment decision-making process are needed.

The aim of our study was to assess whether our 18-gene signature of fibroblast-associated mRNAs, which has shown strong prognostic power for risk of developing distant metastases in human breast cancers [17], was also able to identify CMTs with metastasis formation. While the complete signature did not have any prognostic power in a cohort of 27 canine mammary cancers, a subset of just three mRNAs was able to distinguish CMTs with and without metastases. However, in contrast to the results from the microarray study, RT-qPCR on total RNA isolated from archival FFPE-CMT tissue only showed a significant correlation of *Sfrp1* mRNA with metastasis status. Although *Col13a1* and *Spock2* mRNA abundance showed trends towards up- and downregulation respectively in mammary tumours with metastases, this was not significant and will need to be reassessed in a larger independent cohort. The difference between the microarray study and the RT-qPCR results could be due to the very low level of mRNA abundance measured by our RT-qPCR system for both *Col13a1* and *Spock2* compared to *Sfrp1* mRNA. It is possible that the differences in amplicon sizes of 70 bp (*Sfrp1*), 95 bp (*Spock2*) and 110 bp (*Col13a1*) had a significant impact on the relative detection levels of *Spock2* and *Col13a1*; however, the predicted amplicon of the control *Rps19* (95 bp) was of a similar size. Amplicon location with regard to the 5’ or 3’ end should not have affected the results as we used a random primer/oligo-dT mix for the cDNA synthesis that avoids 3’-5’ bias. Further optimisation of the primer/probe sets to increase sensitivity of the assays is therefore required.

Nevertheless, the RT-qPCR and the IHC data both showed for the first time that SFRP1 expression correlated negatively with Met_pos_ status and tumour grade in CMTs, supporting its potential role as a suppressor of breast cancer progression. Our findings are, however, in contrast to the study by Yu et al. where *Sfrp1* mRNA was significantly upregulated in highly malignant CMTs compared to normal mammary gland tissue [29]. Although our RT-qPCR data did indicate that median *Sfrp1* mRNA abundance was in fact higher in CMTs without metastasis (20.1% of *Rps19*) compared to morphologically normal mammary tissue (8.7%), which would at least partly confirm the data by Yu et al. [29], this did not reach statistical significance and average expression levels were indeed very similar (normal 17.2% vs Met_neg_ 20.1%). However, both morphologically normal and Met_neg_ CMTs had a higher median and average abundance of *Sfrp1* mRNA compared to Met_pos_ CMTs (5.0% and 6.8% respectively). This is consistent with the overwhelming evidence for SFRP1’s role as a tumour suppressor in human breast cancer formation and progression [30–34]. In a study of nearly 2000 breast cancers *Sfrp1* mRNA was down-regulated in nearly 3/4 of invasive breast cancers with unfavourable prognosis in early breast cancer [31]. Similarly, in a comparison of FFPE-sections from human invasive ductal carcinoma (IDC) with ductal carcinoma in situ (DCIS) or morphologically normal control tissue, *Sfrp1* mRNA expression was significantly reduced in IDC [35]. Similarly, gene expression profiling of micro-dissected breast cancer samples from patients with matched IDC, DCIS and associated stroma found that *Sfrp1* mRNA was reduced in the neoplastic epithelium during the progression from DCIS to IDC [36]. Our studies, however, did not detect major SFRP1 protein expression in the neoplastic epithelium. Instead, strong staining of the stromal cells and ECM surrounding the epithelium was observed. Though SFRP1 expression has been found to be reduced in both human primary tumours as well as breast cancer cell lines relative to normal primary human mammary epithelial cells [37], we have not been able to detect *Sfrp1* mRNA in cancerous or normal canine mammary epithelial cell lines by RT-qPCR, but it was strongly expressed in a canine fibroblast cell line (data not shown). Similarly, no SFRP1 was detected by mass spectrometry in total protein of five canine mammary epithelial cell lines (manuscript in preparation). Whether this may represent a species-specific difference is unknown.

One limitation in our study is that we had to use the feline cDNA orthologue to confirm species cross-reactivity and specificity of the monoclonal antibody, which was raised against an unspecified human SFRP1 peptide, as the canine cDNA clone was unavailable (Supplementary Fig. 1B). However, the feline and canine proteins have nearly identical amino acid sequences with only one conservative exchange in position 63, where the canine protein is identical to the human orthologue, and two further variations in positions 15 and 29 of the signal peptide. While position 15 is different in all three species, position 29 is the same in human and cat, but human and feline SFRP1 have a further variance in position 28 so that it’s unlikely that this region was the epitope recognised by the antibody. In addition, the immunohistochemistry pattern for SFRP1 in canine heart muscle resulted in a highly similar staining pattern to the one provided by the manufacturer for the human heart (data not shown). Therefore, a residual risk that this antibody may have recognised the feline but not the canine protein was deemed negligible. As it’s not possible to create a knock-out dog it is of course impossible to fully rule out a potential cross-reactivity with another canine protein in the tissue.

SFRPs are a family of five secreted frizzled-related proteins, all containing a conserved cysteine-rich domain (CRD) with 30–50% sequence homology to the CRD of Frizzled (Fz) receptors. SFRPs are able to bind to Wnt signalling proteins, competing with Fz receptors for their ligands and blocking the canonical Wnt (β-catenin) signalling pathway [38]. SFRPs can further suppress Wnt signalling by forming an inhibitory complex with the Fz receptors themselves [39]. Due to their regulatory ability, SFRP proteins can therefore directly affect Wnt-controlled cell proliferation and differentiation [40].

Although activated canonical Wnt signalling is also linked to poorer overall survival in over 50% of human breast cancers, very few breast cancers harbour somatic mutations. Instead, Wnt ligands, as well as their receptors, are often overexpressed while their antagonists are suppressed [41]. In particular, changes in SFRP expression in cancer tissue has been widely observed, with SFRP1, -3, -4, and -5 commonly downregulated in human breast cancer tissues compared to healthy controls [42]. In humans, loss of Sfrp1 mRNA expression is often achieved through promoter methylation [43]. This is common in primary breast cancers and occurs early during mammary transformation, with SFRP1 expression being consistently lower in atypical hyperplasia compared to histologically normal breast tissue [30]. Here, the reduction in SFRP1 correlates with activated Wnt-signalling [33, 44] while ectopic overexpression of SFRP1 in breast cancer cells blocks Wnt-signalling, decreasing the migratory potential [45] and inhibiting anchorage-independent growth [37]. SFRP1^−/−^ mice also show early tumour initiation as well as an increased tumorigenic potential of SFRP1^−/−^ cancer stem cells (CSC) [46]. This is accompanied by a rise in nuclear β-catenin and Wnt-pathway stimulation.

Recent studies have confirmed that the Wnt-pathway is not just deregulated in human breast cancer but equally in feline and canine mammary cancer compared to healthy mammary tissue [47–49], and that ligand-dependent and independent mechanisms might be involved [47, 48]. Our IHC data also showed strong β-catenin membrane staining in healthy and morphologically normal mammary epithelium and a reduction correlated significantly with tumour progression, which could be indicative of activated canonical Wnt-pathway; however, nuclear β-catenin staining was rare and did not correlate with either progression or SFRP1 expression (Table 3). Further, in vitro studies have shown that β-catenin membrane staining could increase after treatment with recombinant Wnt protein [50], so that a decrease in membranous β-catenin in our tissue may not be a reliable marker for canonical Wnt-pathway activation [27]. Therefore, additional markers, including Axin 2 abundance and/or TCF4 nuclear localisation, will need to be tested to be able to make a clear statement on whether the loss of SFRP1 is associated with canonical Wnt-signalling in CMT or not.

SFRP1 can also affect non-canonical Wnt signalling, including the Wnt-PCP- (planar cell polarity) and Wnt-Ca^2+^-pathway. Recent studies in explant-cultures of mouse and human breast tissue have shown that SFRP1 can further antagonise oestrogen-induced responses, including progesterone receptor (PR) activation [30, 51]. The authors hypothesised that the diminished SFRP1 expression seen in atypical hyperplasia leads to enhanced ER activity and contributes to the development of this premalignant lesion. Though the new 33 CMT cohort had mixed grades and unknown ER/PR status, all cases within our previous cohort of 27 CMTs were higher grade ER_neg_ cancer so that SFRP1 suppression is likely to have an additional role to possibly enhancing ER-signalling in our new cohort. For example, SFRP1 can bind to RANK ligand (RANKL) and thereby inhibit RANK signalling [52], a pathway critical for progesterone-induced mammary epithelial cell proliferation, carcinogenesis and lung metastasis formation [53, 54]. It can therefore not be ruled out that the RANK-pathway may be activated in our SFRP1_low_ CMTs.

Although SFRP1 is downregulated in most breast cancers, this is not the case in the medullary type often associated with triple-negative breast cancers (TNBC), characterised by their lack of hormone receptors ER/PR and HER2. Hence, SFRP1 expression is counter-intuitively often found in younger patients, with higher tumour stage, -size and -grade [55]. However, it is noteworthy that while TNBC are generally more aggressive, the medullary subtype has a better prognosis [56].

In conclusion, our data is consistent with SFRP1 being a potential suppressor of canine mammary tumour progression, and has shown that reduced β-catenin membrane localisation is associated with higher tumour grade and metastasis formation in dogs, but that SFRP1 expression is not significantly associated with β-catenin membrane localisation. Further work is necessary to confirm SFRP1 as a suppressor of mammary tumour progression in the dog similarly to its tumour suppressive role in human breast cancer; to test whether a reduction in SFRP1 leads to an activation of canonical and/or non-canonical Wnt-signalling pathways in canine cancer; and to find out whether CMTs with low SFRP1 may respond to Wnt-inhibiting drug treatment with e.g. SFRP1-mimicking peptides [57].

## Supplementary Information

**Additional file 1: Supplementary Fig. 1.** SFRP1 protein staining pattern in morphologically normal canine mammary tissue. **Supplementary Fig. 2.** Comparison of the SFRP1 mRNA and protein expression levels between individual cases. **Supplementary Fig. 3.** Higher magnification images of β-catenin protein expression in CMTs with and without metastasis. **Supplementary Table 1**. Sequences of primers and fluorescent probes with amplicon sizes and position of probe binding sites that were used for Qpcr. **Supplementary Table 2.** Grading and classification of the cohort of canine mammary gland tumours and morphologically normal canine mammary tissue.

## References

[1] Sleeckx N, de Rooster H, Veldhuis Kroeze E (2011). Canine mammary tumours, an overview. Reprod Domes Anim.

[2] Canadas A, Franca M, Pereira C (2019). Canine mammary tumors: comparison of classification and grading methods in a survival study. Vet Pathol.

[3] Kaszak I, Ruszczak A, Kanafa S (2018). Current biomarkers of canine mammary tumors. Acta Vet Scand.

[4] Goldschmidt M, Pena L, Rasotto R (2011). Classification and grading of canine mammary tumors. Vet Pathol.

[5] DantasCassali G, CavalheiroBertagnolli A, Ferreira E (2012). Canine mammary mixed tumours: a review. Vet Med Int.

[6] Nunes FC, Damasceno KA, de Campos CB (2019). Mixed tumors of the canine mammary glands: evaluation of prognostic factors, treatment, and overall survival. Vet Anim Sci.

[7] Rasotto R, Berlato D, Goldschmidt MH (2017). Prognostic significance of canine mammary tumor histologic subtypes: an observational cohort study of 229 cases. Vet Pathol.

[8] Varallo GR, Gelaleti GB, Maschio-Signorini LB (2019). Prognostic phenotypic classification for canine mammary tumors. Oncol Lett.

[9] Valdivia G, Alonso-Diez Á, Pérez-Alenza D (2021). From conventional to precision therapy in canine mammary cancer: a comprehensive review. Frontiers Vet Sci.

[10] Kaszak I, Witkowska-Pilaszewicz O, Domrazek K (2022). The novel diagnostic techniques and biomarkers of canine mammary tumors. Vet Sci.

[11] Howard B, Ashworth A (2006). Signalling pathways implicated in early mammary gland morphogenesis and breast cancer. PLoS Genet.

[12] Lanigan F, O'Connor D, Martin F (2007). Molecular links between mammary gland development and breast cancer. Cell Mol Life Sci.

[13] Stein T, Price KN, Morris JS (2005). Annexin A8 is up-regulated during mouse mammary gland involution and predicts poor survival in breast cancer. Clin Cancer Res.

[14] Stein T, Salomonis N, Nuyten DSA (2009). A Mouse Mammary Gland Involution mRNA Signature Identifies Biological Pathways Potentially Associated with Breast Cancer Metastasis. J Mammary Gland Biol Neoplasia.

[15] Ibrahim AM, Sabet S, El-Ghor AA (2018). Fibulin-2 is required for basement membrane integrity of mammary epithelium. Sci Rep.

[16] Fata JE, Werb Z, Bissell MJ (2004). Regulation of mammary gland branching morphogenesis by the extracellular matrix and its remodeling enzymes. Breast Cancer Res.

[17] Ibrahim AM, Bilsland A, Rickelt S (2021). A matrisome RNA signature from early-pregnancy mouse mammary fibroblasts predicts distant metastasis-free breast cancer survival in humans. Breast Cancer Res.

[18] Ringnér M, Fredlund E, Häkkinen J (2011). GOBO: Gene Expression-Based Outcome for Breast Cancer Online. PLoS ONE.

[19] Klopfleisch R, Lenze D, Hummel M (2010). Metastatic canine mammary carcinomas can be identified by a gene expression profile that partly overlaps with human breast cancer profiles. BMC Cancer.

[20] Firth D (1993). Bias reduction of maximum-likelihood-estimates. Biometrika.

[21] Heinze G, Schemper M (2002). A solution to the problem of separation in logistic regression. Stat Med.

[22] Detre S, SaclaniJotti G, Dowsett M (1995). A "quickscore" method for immunohistochemical semiquantitation: validation for oestrogen receptor in breast carcinomas. J Clin Pathol.

[23] Stoltzfus JC (2011). Logistic regression: a brief primer. Acad Emerg Med.

[24] Mansournia MA, Geroldinger A, Greenland S (2018). Separation in logistic regression: causes, consequences, and control. Am J Epidemiol.

[25] Kim T-M, Yang IS, Seung B-J (2020). Cross-species oncogenic signatures of breast cancer in canine mammary tumors. Nat Commun.

[26] Geyer FC, Lacroix-Triki M, Savage K (2011). beta-Catenin pathway activation in breast cancer is associated with triple-negative phenotype but not with CTNNB1 mutation. Mod Pathol.

[27] van der Wal T, van Amerongen R (2020). Walking the tight wire between cell adhesion and WNT signalling: a balancing act for beta-catenin. Open Biol.

[28] Misdorp W, Else R, Hellmen E (1999). World Health Organization International Histological Classification of Tumors of Domestic Animals. 2nd Series.

[29] Yu F, Rasotto R, Zhang H (2017). Evaluation of expression of the Wnt signaling components in canine mammary tumors via RT(2) Profiler PCR Array and immunochemistry assays. J Vet Sci.

[30] Gregory KJ, Roberts AL, Conlon EM (2019). Gene expression signature of atypical breast hyperplasia and regulation by SFRP1. Breast Cancer Res.

[31] Klopocki E, Kristiansen G, Wild PJ (2004). Loss of SFRP1 is associated with breast cancer progression and poor prognosis in early stage tumors. Int J Oncol.

[32] Lo PK, Mehrotra J, D'Costa A (2006). Epigenetic suppression of secreted frizzled related protein 1 (SFRP1) expression in human breast cancer. Cancer Biol Ther.

[33] Shulewitz M, Soloviev I, Wu T (2006). Repressor roles for TCF-4 and Sfrp1 in Wnt signaling in breast cancer. Oncogene.

[34] Veeck J, Niederacher D, An H (2006). Aberrant methylation of the Wnt antagonist SFRP1 in breast cancer is associated with unfavourable prognosis. Oncogene.

[35] Dettogni RS, Stur E, Laus AC (2020). Potential biomarkers of ductal carcinoma in situ progression. BMC Cancer.

[36] Vargas AC, McCart Reed AE, Waddell N (2012). Gene expression profiling of tumour epithelial and stromal compartments during breast cancer progression. Breast Cancer Res Treat.

[37] Yang ZQ, Liu G, Bollig-Fischer A (2009). Methylation-associated silencing of SFRP1 with an 8p11-12 amplification inhibits canonical and non-canonical WNT pathways in breast cancers. Int J Cancer.

[38] Dennis S, Aikawa M, Szeto W (1999). A secreted frizzled related protein, FrzA, selectively associates with Wnt-1 protein and regulates wnt-1 signaling. J Cell Sci.

[39] Chim CS, Pang R, Fung TK (2007). Epigenetic dysregulation of Wnt signaling pathway in multiple myeloma. Leukemia.

[40] Uren A, Reichsman F, Anest V (2000). Secreted frizzled-related protein-1 binds directly to Wingless and is a biphasic modulator of Wnt signaling. J Biol Chem.

[41] Zhan T, Rindtorff N, Boutros M (2017). Wnt signaling in cancer. Oncogene.

[42] Wu ZH, Zhang YJ, Yue JX (2020). Comprehensive Analysis of the Expression and Prognosis for SFRPs in Breast Carcinoma. Cell Transplant.

[43] Dahl E, Wiesmann F, Woenckhaus M (2007). Frequent loss of SFRP1 expression in multiple human solid tumours: association with aberrant promoter methylation in renal cell carcinoma. Oncogene.

[44] Cowling VH, D'Cruz CM, Chodosh LA (2007). c-Myc transforms human mammary epithelial cells through repression of the Wnt inhibitors DKK1 and SFRP1. Mol Cell Biol.

[45] Matsuda Y, Schlange T, Oakeley EJ (2009). WNT signaling enhances breast cancer cell motility and blockade of the WNT pathway by sFRP1 suppresses MDA-MB-231 xenograft growth. Breast Cancer Res.

[46] Gauger KJ, Hugh JM, Troester MA (2009). Down-regulation of sfrp1 in a mammary epithelial cell line promotes the development of a cd44high/cd24low population which is invasive and resistant to anoikis. Cancer Cell Int.

[47] Gracanin A, Timmermans-Sprang EP, van Wolferen ME (2014). Ligand-independent canonical Wnt activity in canine mammary tumor cell lines associated with aberrant LEF1 expression. PLoS ONE.

[48] Timmermans-Sprang EPM, Mestemaker HM, Steenlage RR (2019). Dasatinib inhibition of cSRC prevents the migration and metastasis of canine mammary cancer cells with enhanced Wnt and HER signalling. Vet Comp Oncol.

[49] Sammarco A, Gomiero C, Sacchetto R (2020). Wnt/β-Catenin and Hippo Pathway Deregulation in Mammary Tumors of Humans, Dogs, and Cats. Vet Pathol.

[50] Kafri P, Hasenson SE, Kanter I (2016). Quantifying beta-catenin subcellular dynamics and cyclin D1 mRNA transcription during Wnt signaling in single living cells. Elife.

[51] Gregory KJ, Schneider SS (2015). Estrogen-mediated signaling is differentially affected by the expression levels of Sfrp1 in mammary epithelial cells. Cell Biol Int.

[52] Häusler KD, Horwood NJ, Chuman Y (2004). Secreted frizzled-related protein-1 inhibits RANKL-dependent osteoclast formation. J Bone Miner Res.

[53] Gonzalez-Suarez E, Jacob AP, Jones J (2010). RANK ligand mediates progestin-induced mammary epithelial proliferation and carcinogenesis. Nature.

[54] González-Suárez E (2011). RANKL inhibition: a promising novel strategy for breast cancer treatment. Clin Transl Oncol.

[55] Schäfer SA, Hülsewig C, Barth P (2019). Correlation between SFRP1 expression and clinicopathological parameters in patients with triple-negative breast cancer. Future Oncol.

[56] Huober J, Gelber S, Goldhirsch A (2012). Prognosis of medullary breast cancer: analysis of 13 International Breast Cancer Study Group (IBCSG) trials. Ann Oncol.

[57] Mafakher L, Rismani E, Rahimi H (2022). Computational design of antagonist peptides based on the structure of secreted frizzled-related protein-1 (SFRP1) aiming to inhibit Wnt signaling pathway. J Biomol Struc Dyn.

