# Supplementary material for: SFRP1 Expression is Inversely Associated With Metastasis Formation in Canine Mammary Tumours
Source: J Mammary Gland Biol Neoplasia. 2023 Jul 4;28(1):15. doi: 10.1007/s10911-023-09543-z (PMC10319705; doi:10.1007/s10911-023-09543-z)
Supplement: Supplementary file 1 — Additional file 1: Supplementary Fig. 1. SFRP1 protein staining pattern in morphologically normal canine mammary tissue. Supplementary Fig. 2. Comparison of the SFRP1 mRNA and protein expression levels between individual cases. Supplementary Fig. 3. Higher magnification images of β-catenin protein expression in CMTs with and without metastasis. Supplementary Table 1. Sequences of primers and fluorescent probes with amplicon sizes and position of probe binding sites that were used for Qpcr. Supplementary Table 2. Grading and classification of the cohort of canine mammary gland tumours and morphologically normal canine mammary tissue. [file 10911_2023_9543_MOESM1_ESM.docx]

**Supplementary Data:**

**Supplementary Figure 1: SFRP1 protein staining pattern in morphologically normal canine mammary tissue.**

(**A**) Immunohistochemistry for SFRP1 showing strong stromal cell staining of SFRP1 protein surrounding the lobular alveoli and TDLU, together with weaker staining of the associated extracellular matrix. The negative control (neg. ctrl) without primary antibody instead does not show any reactivity. (**B**) Western blot using 25 µg of total protein isolated from canine MTH53A cells transfected with (pCMV-*Sfrp1*) or without (neg. ctrl) a plasmid expressing the feline SFRP1 protein, a nearly identical orthologue, shows the expected specific band of ~40 kDa.

**A**


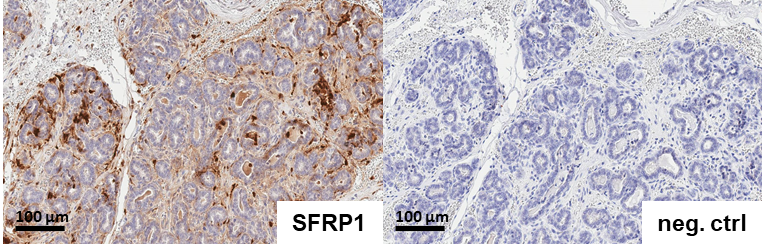


**B**


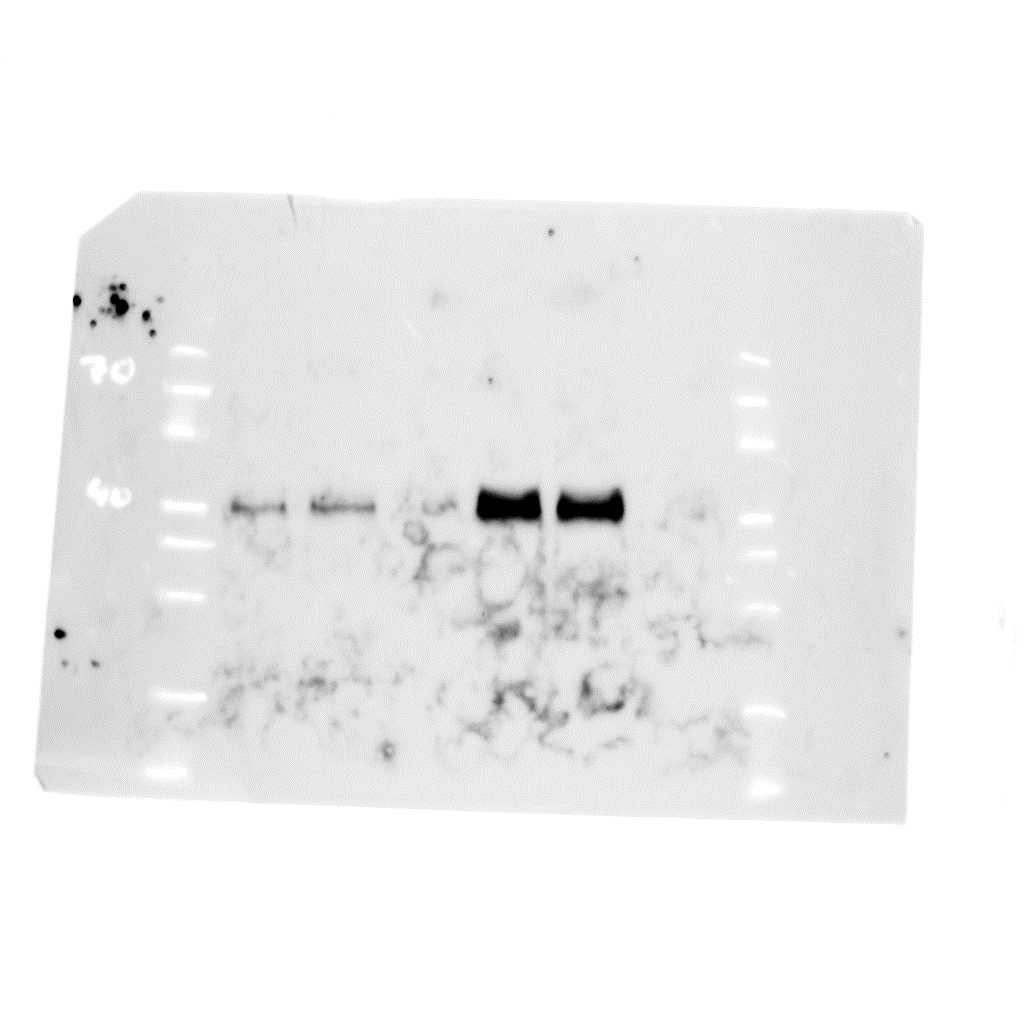


1 µg pCMV-*Sfrp1*

1 µg pCMV-*Sfrp1*

neg. ctrl


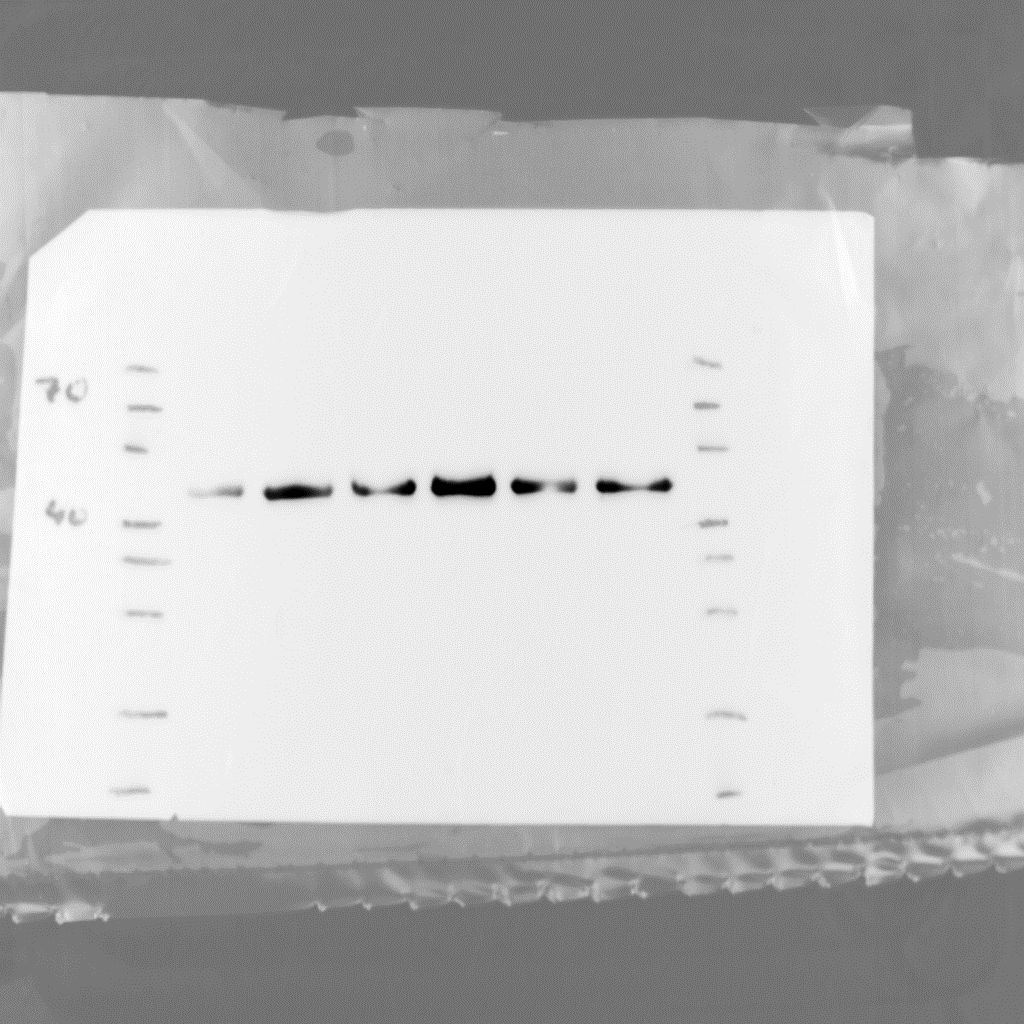


β-actin

SFRP1

1 µg pCMV-*Sfrp1*

1 µg pCMV-*Sfrp1*

neg. ctrl

70 kD

50 kD

40 kD

35 kD

25 kD

15 kD

100 kD

**Supplementary Figure 2: Comparison of the SFRP1 mRNA and protein expression levels between individual cases.**

*Sfrp1* mRNA (as % of *Rps19*) and protein expression levels in metastatic (M1-M17) and non-metastatic (N1-16) CMTs were plotted on a radar chart to compare them on a case-by-case basis (*Sfrp1* mRNA: **0**: <1%; **1**: 1-10%; **2**: 10-25%; **3**: >25% / SFRP1 protein: Quick Score **0** (0), **1** (1-6), **2** (8-12), **3** (>12)). Correlation analysis showed a strong association between *Sfrp1* mRNA and protein levels (p < 0.001).

**
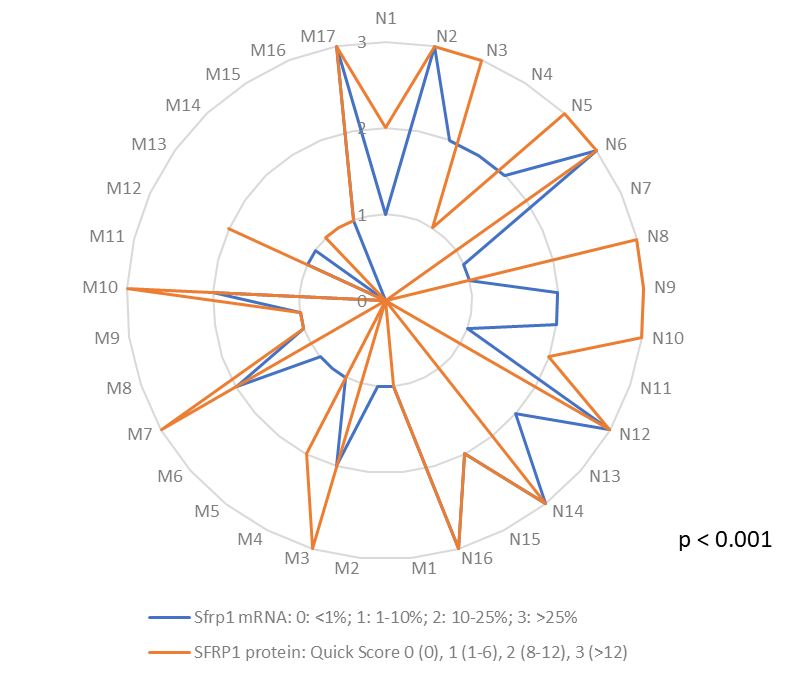
**

**Supplementary Figure 3: Higher magnification images of β-catenin protein expression in CMTs with and without metastasis.**

Higher magnification images of the immunohistochemistry sections stained for β-catenin from cases M1-6 and N1-6 as shown in Figure 3 show the loss of β-catenin membrane staining in most metastatic CMTs. Bars represent 50 µm.

**
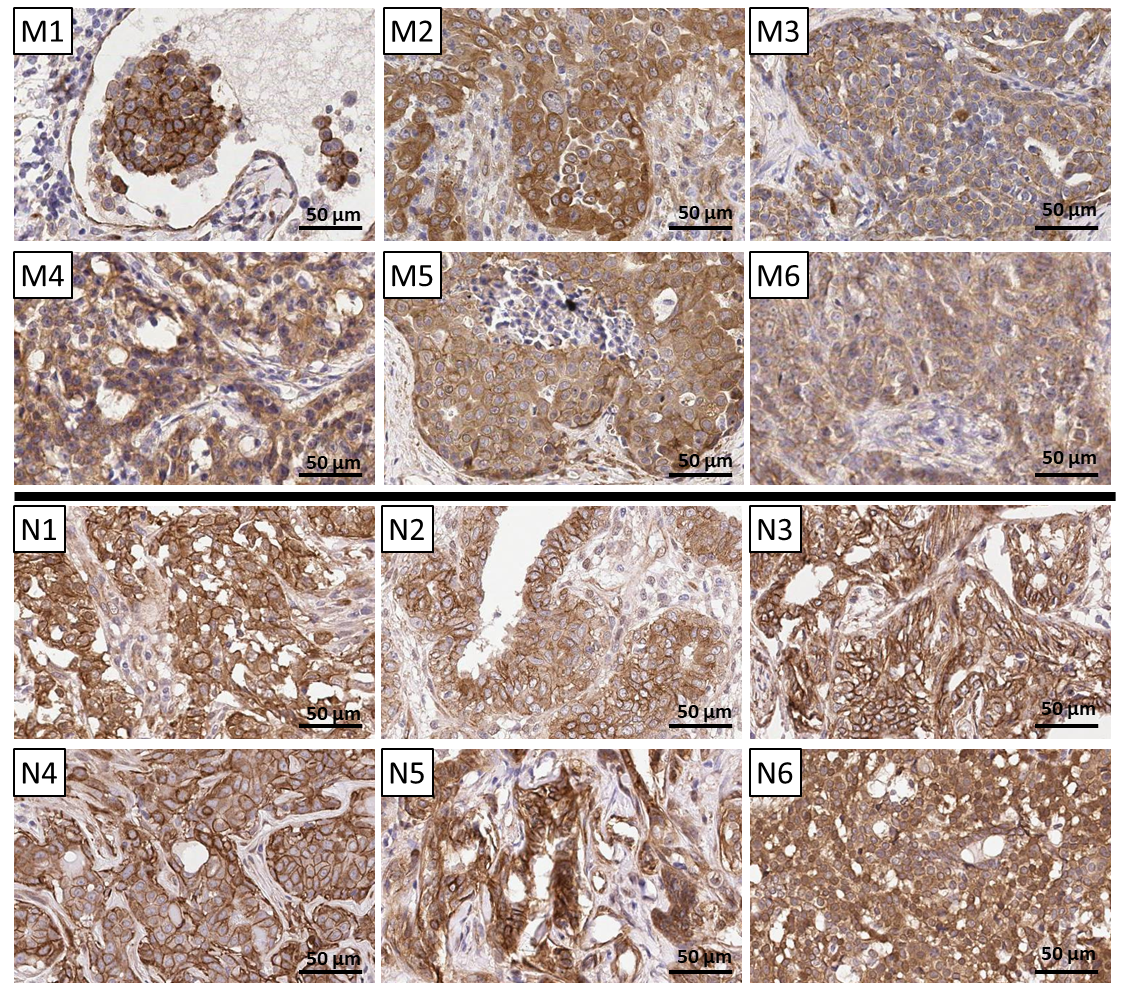
**

**Supplementary Table 1: Sequences of primers and fluorescent probes with amplicon sizes and position of probe binding sites that were used for qPCR**


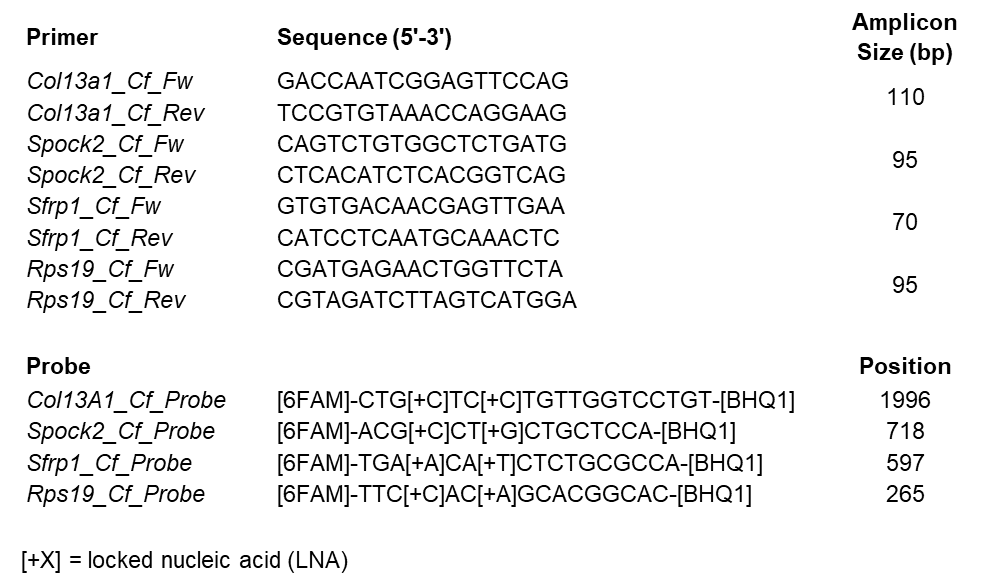


**Supplementary Table 2: Grading and classification of the cohort of canine mammary gland tumours and morphologically normal canine mammary tissue**

| **Year/ Block Nr./**  **M, N or H** | **Grading Points** | **Lymphatic Vessel (LV) Metastasis (pos-neg)** | **Lymph Node (LN) Metastasis (pos-neg).** |
| --- | --- | --- | --- |
| 21/ 4024D/ M1 | 8 | pos | pos |
| 21/ 1621G/ M2 | 8 | pos | pos |
| 21/ 1621D/ M16 | 8 | pos | pos |
| 20/ 1687H/ M4 | 8 | pos | pos |
| 19/ 3977F/ M5 | 8 | pos | pos |
| 19/ 3977A/ M15 | 8 | pos | pos |
| 19/ 2059B/ M14 | 9 | pos | ? |
| 18/ 3093B/ M12 | 9 | pos | pos |
| 17/ 4009-4P/ M11 | 9 | pos | pos |
| 17/ 1859B/ M9 | 8/9 | pos | pos |
| 18/ 7020B/ M13 | 8 | pos | pos |
| 20/ 626A/ N14 | 8 | neg | neg |
| 19/ 2809/ N10 | 8 | neg | neg |
| 19/ 1617/ N9 | 9 | neg | ? |
| 19/ 1857F/ M6 | 6 | pos | pos |
| 20/ 7970B/ M3 | 6 | pos | pos |
| 17/ 3821C/ M10 | 7/8 | pos | ? |
| 17/ 1065-2E/ M8 | 7 | pos | pos |
| 17/ 574/ M7 | 7 | pos | pos |
| 21/ 3386-1B/ M17 | 7 | pos | ? |
| 21/ 4394-1B/ N1 | 7 | neg | neg |
| 20/ 6042-1F/ N6 | 6 | neg | neg |
| 20/ 1952A/ N15 | 6 | neg | neg |
| 19/ 1438C/ N8 | 6/7 | neg | ? |
| 21/ 4022C/ N2 | 5 | neg | neg |
| 21/ 3347B/ N3 | 4 | ? | neg |
| 21/ 900A/ N4 | 5/6 | neg | neg |
| 20/ 8056B/ N5 | 5/6 | neg | neg |
| 20/ 3913F/ N16 | 5 | neg | neg |
| 20/ 274-1C/ N13 | 3 | neg | neg |
| 19/ 5771A/ N12 | 5 | neg | neg |
| 19/ 4326C/ N11 | 5 | neg | neg |
| 19/ 383B/ N7 | 4 | neg | neg |
| 20/ 5300 F/ H1 | na | na | na |
| 16/ 434-2D/ H2 | na | na | na |
| 17/ 63E/ H3 | na | na | na |
| 16/ 6753A/ H4 | na | na | na |
| 17/ 6127A/ H5 | na | na | na |

M: metastatic ? : no lymph vessels or lymph nodes present

N: non-metastatic na: not applicable


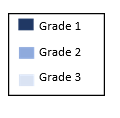
H: healthy tissue
